# Worldwide research productivity in critical care medicine

**DOI:** 10.1186/cc3514

**Published:** 2005-04-04

**Authors:** Argyris Michalopoulos, Ioannis A Bliziotis, Michael Rizos, Matthew E Falagas

**Affiliations:** 1 Director, Intensive Care Unit, Henry Dunant Hospital, Athens, Greece; 2 Research fellow, Alfa Health Care, Athens, Greece; 3 Attending Physician, Intensive Care Unit, Henry Dunant Hospital, Athens, Greece; 4 President, Board of Trustees, Alfa Institute of Biomedical Sciences (AIBS), Athens, Greece, and Adjunct Assistant Professor of Medicine, Department of Medicine, Tufts University School of Medicine, Boston, Massachusetts, USA

## Abstract

**Introduction:**

The number of publications and the impact factor of journals are accepted estimates of the quantity and quality of research productivity. The objective of the present study was to assess the worldwide scientific contribution in the field of critical care medicine.

**Method:**

All research studies published between 1995 and 2003 in medical journals that were listed in the 2003 Science Citation Index (SCI^®^) of Journal Citation Reports under the subheading 'critical care' and also indexed in the PubMed database were reviewed in order to identify their geographical origin.

**Results:**

Of 22,976 critical care publications in 14 medical journals, 17,630 originated from Western Europe and the USA (76.7%). A significant increase in the number of publications originated from Western European countries during the last 5 years of the study period was noticed. Scientific publications in critical care medicine increased significantly (25%) from 1995 to 2003, which was accompanied by an increase in the impact factor of the corresponding journals (47.4%). Canada and Japan had the better performance, based on the impact factor of journals.

**Conclusion:**

Significant scientific progress in critical care research took place during the period of study (1995–2003). Leaders of research productivity (in terms of absolute numbers) were Western Europe and the USA. Publications originating from Western European countries increased significantly in quantity and quality over the study period. Articles originating from Canada, Japan, and the USA had the highest mean impact factor.. Canada was the leader in productivity when adjustments for gross domestic product and population were made.

## Introduction

Critical care is an integral part of hospitals, consuming an important proportion of all beds and of the hospital budget. Critical care medicine is thought to account for 1% of the gross domestic product (GDP) in the USA and has been implicated in a disproportionate amount of the increase in hospital costs [1-3]. In a recently reported study conducted over a 16-year period, Halpern and coworkers [4] demonstrated that, 'Critical Care Medicine is increasingly used and prominent in a shrinking U.S. hospital system'. In addition, during recent decades there was increasing utilization of intensive care unit (ICU) resources by the elderly. Although adults younger than 65 years accounted for 37 ICU days/year per 1000 population, patients aged 65–85 years incurred five to six times that rate [5].

Intensive care medicine is a unique discipline. It is practised by physicians from several primary specialties, all having special training in emergency and critical care medicine. Research is an important and special field that intensivists all over the world deal with, in addition to their daily clinical practice. Publications represent a central part of the research process.

The objective of this bibliometric analysis was to examine the geographical origin of biomedical publications in the area of critical care medicine. We also examined the quantity and quality of these publications from around the world.

## Methods

### Journals

All journals in the 'critical care medicine' category of the Journal Citation Reports (JCR) database, according to the Institute for Scientific Information [6], within the period 1995–2003 were included. To identify whether these journals were included in Index Medicus, we performed a detailed computerized search for each journal in PubMed's database for every year of the study period [7]. Journals included in the 'critical care medicine' category of the JCR database but not indexed in Index Medicus were excluded from the study. We also excluded medical journals referring to emergency medicine and all nursing journals dealing with critical care issues. We did not include articles published before 1995 because the full address of the authors was frequently not registered in PubMed prior to this year. Furthermore, the JCR database provided available data up to the year 2003 at the time of our data collection.

To quantify research productivity, the number of published articles was considered an index of quantity. The mean impact factor of the published articles was considered an indicator of quality. Finally, the product of the number of articles published in a journal multiplied by the impact factor of the journal, for each year studied, was considered a combined indicator of the quantity and quality of research productivity. The sum of the above products from all journals, for each world region within a year, was considered a 'total product' for that region.

### Search procedures

A phrase consisting of four parts joined together by the so-called Boolean operators (i.e. AND, OR, and NOT) was used in our search of the PubMed database. Each search was limited to a specific year using the 'Limits' option, which is incorporated into the search engine. We only analyzed data from original articles and reviews, excluding publication types such as letters, editorials and news reports. This was accomplished by selecting publications of type 'journal article [pt]' in the search field of the database ('pt' means publication type). For example, in order to search for articles published in 'intensive care medicine' originating from Europe, the following phrase was used (where 'AD' means 'address'): intensive care medicine [journal] AND journal article [pt] AND (Andorra [AD] OR Austria [AD] OR ... OR Wales [AD]) NOT (Australia [AD] OR Canada [AD] OR ...). Included were all countries from each region cited in the first pair of parenthesis of the search phrase. The second pair of parenthesis, following 'NOT', contained countries to be excluded in order to avoid double counting.

The results of our search (the number of articles produced by each world region in a specific journal within a year) were summed. We confirmed our findings by summing the number of articles retrieved in our search for all different world regions in a specific journal and comparing the sum with the actual total number of articles published in the same journal for a specific year. The total number was obtained from PubMed without using address limits. Using this methodology, we were able to cross examine those articles for which the originating location was either missing or not retrieved in our search. This scenario did occur occasionally, where articles had no registered address or only the affiliated institution or the city (and not the country) was recorded.

If fewer than 5% of the total articles from a specific journal during 1 year had missing/unretrieved addresses, we considered the number of articles retrieved from the search sufficient. On the other hand, if more than 5% of the total articles from a specific journal during 1 year had missing addresses, we performed additional searches for the author's address by checking other articles from the same author within the same year. In order to include addresses for which only cities or areas were registered, we expanded our search criteria, including search phrases with large cities or capitals (e.g. Munchen, London or Moscow) and all of the individual states of the USA.

Using this retrieval system we identified a few addresses that were double-counted in two different regions. For instance, if 'Beth Israel' – the name of several hospitals in the USA – appears in the address field of the article, then this individual article could be counted, for example, in both USA and Asia. To avoid such problems, a large number of initial search results was manually checked and exclusion criteria were added in the second parenthesis of this search string; for example, when searching for Asia, we added the following: NOT (Beth Israel [AD] OR USA [AD]). Two investigators from our team performed independent searches to further strengthen our methodological validity. In cases of disagreement between the two investigators, the findings were discussed at meetings including all authors and final decisions were based on majority consensus.

### World regions

For the purposes of the present study, the world was divided into nine regions based on a combination of geographic, economic and scientific criteria [8].: Western Europe, USA, Japan, Canada, Asia, Eastern Europe, Oceania, Latin America and the Caribbean, and Africa. All former socialist countries of Europe and Turkey were included in the category of Eastern Europe. Greenland was designated Western Europe. Japan was studied as a separate region relative to the rest of Asia. Puerto Rico and the Virgin Islands were included within the USA region.

### Relationships of research productivity with economic and scientific resources

The relevant 'World Development Indicators' from the online databases of the World Bank were used for further evaluation of the association between research productivity of each region and other factors [9]. The research productivity of different world regions (estimated by the 'total product') was evaluated in relation to the total population, GDP in standard 1995 US dollars and gross national income (GNI) per capita (Atlas method). Data analysis was performed using statistical software SPSS 10.0., SPSS Inc., 233 S. Wacker Drive, Chicago, Illinois 60606, USA.

## Results

Of 26 journals related directly to the field of critical care medicine, 16 were listed in the 2003 Science Citation Index (SCI^®^) of the JCR database under the subheading 'critical care'. Of these, 14 were also indexed in the PubMed database. The titles of these medical journals are presented in Table 1.

A total of 23,403 articles published in journals included in the 'critical care' category of the JCR database and indexed in PubMed within the period 1995–2003 were evaluated in the study. We were able to retrieve 98.2% of all articles (22,976 articles) and categorize them according to the country of origin, based on the methodology described above. Table 2 shows the number of studies originating from each world area/year within the period 1995–2003. In addition, the total number of publications by world region and the relative contribution of each region to the total production of articles, for all journals retrieved, are also presented. The majority of articles published between 1995 and 2003 originated from Western Europe and the USA (76.7%). More articles originated from Western Europe than from USA during the last 6 years of the study period. The USA ranks second, except in years 1995–1997, when production from the USA exceeded that from Western Europe. Asia (excluding Japan) ranks third, Canada fourth, Oceania fifth, and Japan sixth. Eastern Europe, Central and Latin America, and Africa made little contribution in critical care research within this period. A significant increase in the number of publications originating from Western European countries during the last 5 years of the study period was noticed.

Although more articles originated from Western European countries than from the other world regions, the mean impact factor for articles from Western Europe over the study period was lower than the mean impact factors for articles originating from the USA, Canada and Japan (Table 3). Among the regions studied, publications from Eastern Europe had the lowest mean impact factor.

Table 4 presents the 'total product' (summation of [number of published articles in a journal × the impact factor of that journal] for all journals included) for each world region. The USA had the greatest total product, Western Europe ranked second and was followed by Canada, Japan, Asia (excluding Japan) and Oceania. Eastern Europe, Central and Latin America, and Africa made little contribution. Western Europe and the USA exhibited the most significant relative growth in the total product of medical research in the field of critical care over the period 1995–2003, followed by Canada Japan, Asia and Oceania. All other regions exhibited minimal growth in research productivity.

Table 5 presents the total product adjusted for regional population as well as the GDP of the studied regions. Canada ranks first among the world areas with respect to production adjusted for both variables. Oceania also ranks high (second when production was adjusted for GDP and third when it was adjusted for population). USA outweighs Western Europe for both adjustments.

**Figure 1:**
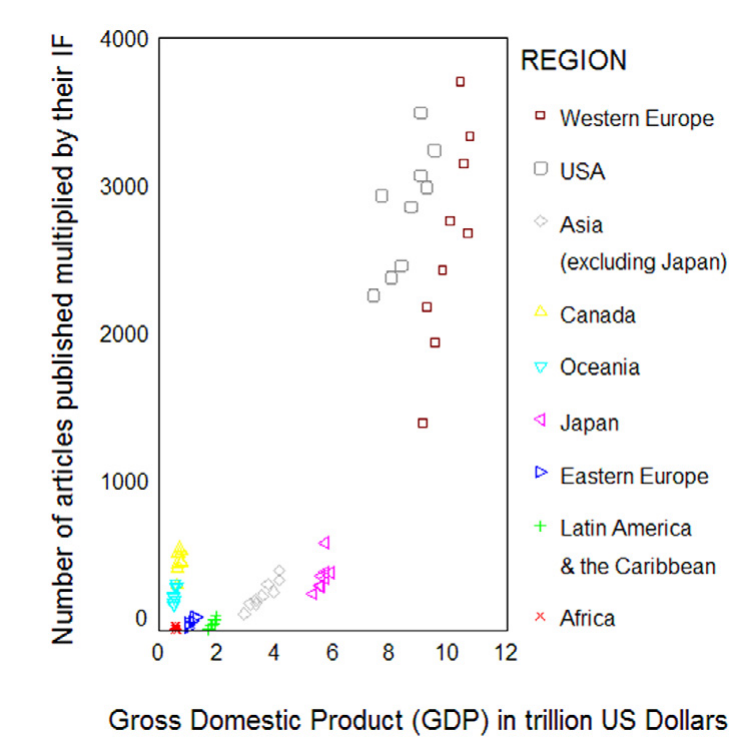
'Total product' of research productivity. Scatter plot depicting the relationship of the annual total product of research productivity (number of articles published × their impact factor [IF]) in critical care medicine, for different world regions, for the period 1995–2003, with the gross domestic product (GDP) in trillions of 1995 US dollars.

In Fig. 1 we present the association between the GDP in trillions of 1995 US dollars and the 'total product' of research for each region. For all regions there is a positive association between GDP and total product. Publication performance of Canada, Oceania, Western European countries and the USA was better in relation to quantity and quality of articles as compared with the other regions.

**Figure 2:**
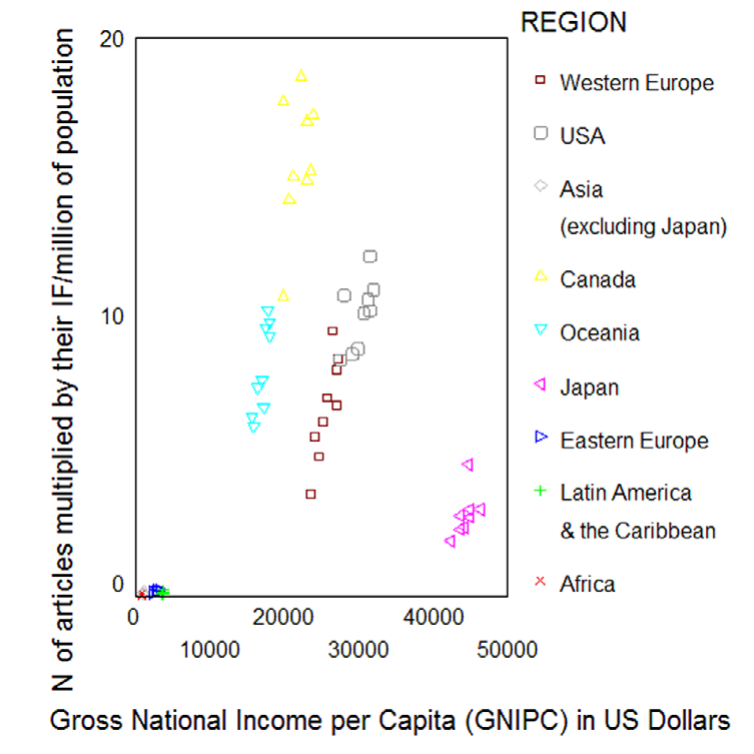
Relation of 'total product' adjusted for population to GNIPC. Scatter plot depicting the relationship of the annual "total product" of research productivity for different world regions (number of articles published multiplied by their impact factor) in Critical Care Medicine, with the population of a region and the gross national income per capita (GNIPC)

Figure 2 shows the association between GNI per capita and the total product of research adjusted for population size for each region. The regions are clustered into three groups. Africa, Asia, Eastern Europe, and Central and Latin America comprise the first group, in which both GNI per capita and total product adjusted for population size are very low. The second group consists of Canada, USA, Oceania and Western Europe, in which the greater the GNI per capita, the greater the population-adjusted total product. Japan stood out as an example of high GNI per capita associated with relatively lower population adjusted total product.

## Discussion

Following our evaluation of worldwide trends in research productivity in the field of critical care medicine research over a 9-year period (1995–2003), we conclude that Western Europe produces the most reports on critical care medicine. Western Europe is the only region around the world exhibiting a significant absolute increase in research productivity over the period studied. Nevertheless, although USA produced fewer publications than did Western Europe in this field, the mean impact factor of the published articles from the USA was higher (3.01 versus 2.60). It is remarkable that publications in journals with higher mean impact factors originated from Canada, Japan and Latin America. Although the value of the impact factor as a tool for assessing the quality of a medical journal is controversial, publications in critical care journals from all world areas showed a significant increase in their mean impact factor over this period. However, it should be noted that the average impact factors for anaesthesia and critical care journals, as well as those for other biomedical journals, have tended to increase over recent years for several reasons [10,11].

Scientific publications in critical care medicine increased significantly (25%) from 1995 to 2003, which was accompanied by increased impact factors for these journals (47.4%). Subsequently, the product of the number of published articles multiplied by the impact factor of each journal ('total product') – a combined indicator of research productivity – also increased within the study period. Western Europe and USA together produced 76.7% of the total number of articles published in the field of critical care medicine. These two world regions were superior to all others in terms of total research productivity. It is clear that scientific productivity from these two world regions in this new discipline has increased exponentially over the period of study. In contrast, the contributions of other world regions to research productivity were low, especially those from low income areas such as Eastern Europe, Latin America and the Caribbean, and Africa. This might be because critical care medicine was mainly developed in countries with vigorous economies, because the cost of hospitalization in ICUs is high. It should be emphasized that several factors, including resources, interest in research and language barriers, influence research productivity by various areas of the world.

When total product was adjusted for GDP and/or regional population, Canada ranked first and Oceania ranked very high. Thus, these two regions are clearly among world leaders in research in this field, but because of their relatively small populations (and consequently relatively small GDPs) their absolute number of publications is small. In two previous studies that we conducted using the same methodology, one in the 'Cardiac and cardiovascular systems' category [12] and one in the 'Microbiology' category [13] of the JCR database, the results were similar to those of the present study. Again, Western Europe and USA were the leaders in terms of absolute number of papers, whereas Canada and Oceania were in the top positions when adjustments for GDP and population were made.

Although intensivists from the USA led the research in critical care medicine, their colleagues from Western European countries made greater contribution during the last 6 years of study. It is noteworthy that North America and Canada performed better than Europe in terms of mean impact factor of publications. Similar findings were reported for fields other than critical care (i.e. cardiology, clinical cancer, microbiology and radiology) [12-16].

We should like to acknowledge several limitations of this study. First, we used JCR criteria for inclusion of medical journals in the present study. Articles published in non-JCR cited journals were not included, but we do recognize that they contribute to scientific production [17]. This pertains in particular to originating regions in which English is not the native language (i.e. Eastern Europe and Japan), where researchers tend to publish their findings in regional journals of their own language [18]. We also used Medline, which is an easily accessible and widely used database. It should also be emphasized that, in Medline, only the address of the first author is presented; that a study might be the result of multinational collaboration is not taken into account. Furthermore, it is known that there are many medical journals on critical care medicine from all over the world in languages that are not indexed.

In addition, one should take into account that the impact factor, as an index of quality of scientific research, has often been criticized [19,20]. Impact factors change every 12 months, and so they are not very responsive to change. However, the impact factor is yet to be replaced by another internationally accepted method [21]. Furthermore, the division of the world into regions could be done in several different ways, based on various criteria (e.g. Canada could be grouped with the USA, and Japan could be studied together with the rest of Asia). We believe that our categorization takes into account geographic, economic and, most importantly, scientific criteria (i.e. Canada and Japan represent powerful scientific world regions on their own).

Finally, when interpreting the results, one should take into account the fact that many articles regarding critical care medicine are published in journals other than those included in the 'critical care medicine' category. Furthermore, a proportion of articles published in journals included in the 'critical care medicine' category of the Science Citation Index are related to non-critical-care topics. However, it seems that there is no systematic bias in the analysis of these data, because there is no specific reason to publish articles on this subject in journals included in other JCR categories, especially from specific world regions, and neither is there any reason for non-critical-care articles to originate mainly from certain regions.

## Conclusion

We took a global view of worldwide trends in research productivity in the field of critical care medicine over a 9-year period. It is notable that Western Europe and USA ranked top in terms of quantity and quality of published articles in absolute numbers, whereas Canada was the leader in productivity when adjustments for GDP and population were made. As expected, developed world regions ranked first in quantity and quality of published articles, and had greater productivity adjusted for population.

Our data may be used to compare the productivity of different world regions with diverse economic status and priorities for funding different social needs. The World Health Organization, the World Bank, other United Nations organizations and national governments should encourage biomedical research in less developed parts of the world. Intensivists all over the world must acquire and maintain the necessary skills to provide state-of-the art clinical care for critically ill patients so that they may confront life-threatening disease, improve patient outcomes, optimize the use of limited ICU resources and, in parallel, advance the theory and practice of critical care medicine. The quality of care provided in ICUs worldwide has improved over the past decade. Nevertheless, disorders such as adult respiratory distress syndrome, sepsis and ICU-acquired infections remain foci of interest, and are difficult to manage and associated with high mortality rates. Consequently, further research studies on several fields are urgently needed.

## Competing Interests

The author(s) declare that they have no competing interests.

## Key messages

• 	Leaders of research productivity in critical care medicine, in terms of absolute numbers of published papers during the study period (1995–2003), were Western Europe and the USA.

• 	Articles originating from Canada, Japan, and the USA had the highest mean impact factor.

• 	Canada was the leader in productivity when adjustments for gross domestic product and population were made.

## Abbreviations

GDP = gross domestic product; GNI = gross national income; ICU = intensive care unit; JCR = Journal Citation Reports.

## Authors' contributions

AM and MEF conceived the study. IAB and MR collected data. All authors contributed to the writing and preparation of the manuscript.

## Figures and Tables

**Table 1:** Summary of journals related to critical care medicine included in the study

Abbreviated journal title	Impact factor (2003)
*Am J Resp Crit Care*	8.87
*Crit Care Med*	4.19
*Intensive Care Med*	2.97
*J Neurotrauma*	2.58
*Shock*	2.54
*Crit Care*	1.91
*Crit Care Clin*	1.48
*J Trauma*	1.42
*Resuscitation*	1.37
*J Crit Care*	1.29
*Burns*	1.12
*Anaesth Intens Care*	0.77
*Injury*	0.51
*Anasth Intensiv Notf*	0.31

**Table 2:** Number of articles published in critical care medicine: 1995–2003

World area	Year(s)									
	
	1995	1996	1997	1998	1999	2000	2001	2002	2003	1995–2003
Western Europe	720 (32.7)	829 (35.2)	801 (36.3)	870 (38.6)	1037 (41.3)	1324 (43.2)	1223 (43.1)	1125 (40.2)	1147 (41.7)	9076 (39.5)
USA	982 (44.6)	978 (41.5)	853 (38.6)	829 (36.8)	904 (36.0)	1088 (35.5)	992 (35.0)	972 (34.8)	956 (34.8)	8554 (37.2)
Asia (excluding Japan)	114 (5.2)	128 (5.4)	132 (6.0)	134 (6.0)	138 (5.5)	154 (5.0)	169 (6.0)	193 (6.9)	186 (6.8)	1348 (5.9)
Canada	113 (5.1)	144 (6.1)	126 (5.7)	126 (5.6)	139 (5.5)	134 (4.4)	123 (4.3)	129 (4.6)	130 (4.7)	1164 (5.1)
Oceania	120 (5.5)	116 (4.9)	128 (5.8)	123 (5.5)	111 (4.4)	120 (3.9)	128 (4.5)	140 (5.0)	130 (4.7)	1116 (4.9)
Japan	94 (4.3)	92 (3.9)	103 (4.7)	107 (4.8)	110 (4.4)	173 (5.6)	120 (4.2)	126 (4.5)	101 (3.7)	1026 (4.5)
Eastern Europe	24 (1.1)	39 (1.7)	29 (1.3)	28 (1.2)	35 (1.4)	30 (1.0)	33 (1.2)	61 (2.2)	46 (1.7)	325 (1.4)
Latin America and the Caribbean	8 (0.4)	13 (0.6)	16 (0.7)	19 (0.8)	21 (0.8)	29 (0.9)	31 (1.1)	35 (1.3)	34 (1.2)	206 (0.9)
Africa	25 (1.1)	19 (0.8)	20 (0.9)	16 (0.7)	13 (0.5)	15 (0.5)	17 (0.6)	16 (0.6)	20 (0.7)	161 (0.7)
										
Total	2200 (100)	2358 (100)	2208 (100)	2252 (100)	2508 (100)	3067 (100)	2836 (100)	2797 (100)	2750 (100)	22976 (100)

Shown are the numbers of articles published in journals included in the 'critical care medicine' category of the Journal Citation Report database and indexed by PubMed, from different world regions, for the period 1995–2003. Values are expressed as number of articles (%) within a calendar year.

**Table 3:** Mean impact factors of articles published in critical care medicine: 1995–2003

World area	Year									
	
	1995	1996	1997	1998	1999	2000	2001	2002	2003	1995–2003
Western Europe	1.95	2.64	2.43	2.80	2.67	2.80	2.58	2.39	2.91	2.60
USA	2.30	3.01	2.80	2.98	3.16	3.21	3.09	3.07	3.39	3.01
Asia (excluding Japan)	1.10	1.49	1.44	1.57	1.72	2.06	1.59	1.76	2.19	1.70
Canada	2.79	3.66	3.38	3.62	4.10	3.92	3.77	3.71	4.20	3.70
Oceania	1.53	1.53	1.74	1.90	1.87	2.46	2.50	2.21	2.31	2.01
Japan	2.65	3.26	2.99	2.82	3.33	3.47	3.00	3.13	3.93	3.19
Eastern Europe	0.86	1.46	1.09	1.56	1.44	2.00	1.83	1.43	1.99	1.55
Latin America and the Caribbean	2.42	3.30	2.23	2.85	2.68	2.65	2.57	2.23	3.11	2.66
Africa	0.98	2.29	1.75	1.52	2.06	1.16	1.26	1.08	2.11	1.57
										
Mean (for all regions)	2.09	2.74	2.53	2.76	2.85	2.97	2.75	2.64	3.08	2.73

Shown are the mean impact factors of articles published in journals included in the 'critical care medicine' category of the Journal Citation Report database and indexed by PubMed, from different world regions, for the period 1995–2003.

**Table 4:** 'Total product' of articles published in 'critical care medicine': 1995–2003

World area	Year									
	
	1995	1996	1997	1998	1999	2000	2001	2002	2003	1995–2003
Western Europe	1401	2186	1944	2434	2772	3713	3158	2694	3340	23,642
USA	2263	2941	2390	2468	2860	3492	3068	2989	3244	25,715
Asia (excluding Japan)	125	191	190	211	237	317	269	341	407	2288
Canada	316	526	426	456	570	525	464	479	546	4308
Oceania	184	178	222	233	207	295	320	310	300	2249
Japan	249	300	308	302	367	600	360	394	397	3276
Eastern Europe	21	57	32	44	50	60	61	87	91	502
Latin America and the Caribbean	19	43	36	54	56	77	80	78	106	549
Africa	24	44	35	24	27	17	21	17	42	252
										
										
Total	4602	6464	5583	6226	7147	9096	7800	7389	8473	62781

Shown are the total products of articles (number of articles published × their impact factor) published in journals included in the 'critical care medicine' category of the Journal Citation Report database and indexed by PubMed, from different world regions, for the period 1995–2003.

**Table 5:** Product of number of articles and impact factor, adjusted for population and GDP

World Areas	Number of articles multiplied by their impact factor/population of the area (in millions)	Number of articles multiplied by their impact factor/GDP of the area (in hundreds of billions 1995 US dollars)
Western Europe	6.74	26.3
USA	10.10	33.5
Asia (excluding Japan)	0.07	7.1
Canada	15.67	71.5
Oceania	8.15	47.9
Japan	2.88	6.5
Eastern Europe	0.13	5.1
Latin America and the Caribbean	0.12	3.2
Africa	0.04	5.2

Shown are the numbers of articles in journals included in the 'critical care medicine' category of the Journal Citation Report database multiplied by their impact factors, adjusted for population and gross domestic product (GDP).
